# Senile dementia and pharmacological drugs

**DOI:** 10.1590/S1980-57642013DN70200007

**Published:** 2013

**Authors:** Milton Luiz Gorzoni, Renato Moraes Alves Fabbri, Sueli Luciano Pires

**Affiliations:** 1 Associate Professor. Department of Internal Medicine of the Faculty of Medical Science of the Santa Casa de São Paulo Hospital (DCM-FCMSCSP), São Paulo SP, Brazil.; 2 Assistant Professor. Department of Internal Medicine of the Faculty of Medical Science of the Santa Casa de São Paulo Hospital (DCM-FCMSCSP), São Paulo SP, Brazil.; 3 Teaching Professor. Department of Internal Medicine of the Faculty of Medical Science of the Santa Casa de São Paulo Hospital (DCM-FCMSCSP), São Paulo SP, Brazil.

**Keywords:** elderly, senile dementia, alzheimer type, pharmaceutical preparations, iatrogenic disease

## Abstract

Side effects and drug interactions are common in the elderly and highly relevant in the demented, being routinely confused with symptoms of cognitive impairment. Which drugs are most consumed by this patient group? Do prescription patterns differ between the demented and non-demented?

**OBJECTIVE:**

To define drug consumption quantitatively and qualitatively in demented (D) and non-demented (ND) elderly.

**METHODS:**

Patients were divided into men and women, by age group (<80 and ≥80 years), non-demented and demented status, and consumers of ≤3 or >3 drugs. As a criterion comparing groups, the Chi-square (Fisher's exact) test was employed. This study is part of Project No. 405/10 approved by the Ethics Committee of the institution.

**RESULTS:**

The sample had a mean age of 81.5±8.8 years, 29 D (21 women and 8 men) and 21 ND (16 women and 5 men), 12 consumers of up to three drugs (7 D and 5 ND) and 38 consumers of 3 medications or more (22 D and 16 ND). The most used drugs among dementia patients were aspirin, angiotensin-converting enzyme inhibitors, statins, selective serotonin reuptake inhibitors, and vitamins. Drugs most consumed by non-demented included vitamins, aspirin, calcium carbonate, proton pump inhibitors, statins and alendronate sodium. There was no statistical significance on any of the comparisons, although the number of elderly consumers of vitamins in the ND had a p-value of 0.06 (Yates).

**CONCLUSION:**

The elderly in this series, regardless of dementia status, gender or age group, had similar drug consumption patterns and used multiple drugs simultaneously.

## Abstract (pt)

Iatrogenias, efeitos colaterais e interações medicamentosas são comuns em idosos e altamente relevantes em dementados, onde corriqueiramente as manifestações decorrentes são confundidas com disfunção cognitiva. Quais seriam os medicamentos mais consumidos por esses pacientes? O padrão de prescrição diferiria dos não-dementados?

**OBJETIVO:**

Definir os padrões de consumo medicamentoso, quantitativos e qualitativos em dementados (D) e não-dementados (ND).

**MÉTODOS:**

Casuística dividida entre homens e mulheres, por idade (<80 e ≥80 anos), dementados e não-dementados, consumidores de ≤3 ou >3 medicamentos. Como critério comparativo entre grupos, utilizou-se qui-quadrado (teste exato de Fisher). Esse estudo faz parte do Projeto No. 405/10 aprovado pelo Comitê de Ética em Pesquisa da Irmandade da Santa Casa de Misericórdia de São Paulo.

**RESULTADOS:**

A Idade média foi de 81,5±8,8 anos, 29 D (21 mulheres e 8 homens) e 21 ND (16 mulheres e 5 homens), 12 consumidores de até 3 medicamentos (7 D e 5 ND) e 38 consumidores de mais de 3 medicamentos (22 D e 16 ND). Os medicamentos mais consumidos entre os dementados foram: ácido acetil salicílico, inibidores da enzima conversora da angiotensina, estatinas, inibidores da recaptação da serotonina e vitaminas. Fármacos mais consumidos entre os não-dementados: vitaminas, ácido acetil salicílico, carbonato de cálcio, inibidores da bomba de prótons, estatinas e alendronato de sódio. Não houve significância estatística nas comparações entre os grupos D e ND, embora o número de idosos ND consumidores de vitaminas apresente p 0,06 (Yates).

**CONCLUSÃO:**

Na presente casuística, dementados ou não, independentemente do gênero ou da faixa de idade, apresentam padrão medicamentoso semelhante, merecendo ênfase a multiplicidade de fármacos em uso simultaneamente.

## INTRODUCTION

Side effects and drug interactions are common in the elderly^1-5^ and highly relevant in the demented, where they are often confused with symptoms of cognitive impairment^6-9^ This problem occurs frequently in ide effects and drug interactions are com-routine clinical practice, particularly involvmon in the elderly^1-5^ and highly relevant ing drugs unrelated to specific treatment for in the demented, where they are often con-dementia conditions. A search carried out on fused with symptoms of cognitive impair-21/07/2012, on the portal http://www.scielo.br, with the key words "dementia" and "senile" associated with "comorbidities" or "medications" retrieved no scientific articles on the topic of general medications and demented elderly. The dearth of studies in the literature precluding a review of this issue prompted the present study whose aim was to analyze use of medications not associated with the pharmacological treatment of the dementia syndrome.

With the aim of assessing the consumption of medications not specifically for dementia in demented elderly on their first appointment at the Geriatrics outpatient clinic, the present study was centered on the following two questions: which drugs are most consumed by these patients? Do prescription patterns differ between demented and non-demented groups?

## METHODS

An analysis was performed based on medical records from patients' first appointment at the Geriatrics outpatient clinic. This approach was elected owing to the virtual absence of specific medications for treatment of dementia conditions at this specific appointment, given the clinic in question is responsible for dispensing high-cost drugs, particularly anticholinergic agents.

Participants were divided into two sub-groups:

1. Demented Group (D) - aged 60 years or older and diagnosed with senile dementia syndrome according to DSM-IV criteria.^10,11^
2. Non-demented Group (ND) - aged 60 years or older and not diagnosed with senile dementia syndrome according to DSM-IV criteria.^10,11^

The qualitative and quantitative assessment divided the two groups as follows: [1] men and women; [2] age (under 80 and 80 years or over); and [3] patients reporting use of up to three drugs or three or more medications at entry anamnesis.

The qualitative assessment analyzed potentially inappropriate medications (PIMs) for older adults - defined as drugs having side effect risks that outweigh their benefits in the elderly - independently of diagnosis or clinical condition, based on the Beers-Fick criteria (2003 version)^5^ adapted for the Brazilian Pharmacopeia (Table 1).^12^ The two subgroups were further subdivided into: [1] men and women; [2] age (under 80 and 80 years or over); and [3] consumers and non-consumers reporting one or more PIM for older adults at entry anamnesis.

**Table 1:** Potentially inappropriate medications in older adults, independently of diagnoses or clinical condition, given their high risk of side effects and commercial availability of safer alternatives in Brazil,^12^ according to the Beers-Fick criteria (2003 version).^5^

Benzodiazepines	• Lorazepam >3.0 mg/day		Amiodarone	
	• Alprazolam >2.0 mg/day		Digoxin >0.125 mg/day (except in atrial arrhythmias)	
	• Chlordiazepoxide		Disopyramide	
	• Diazepam		Methyldopa	
	• Clorazepate		Clonidine	
	• Flurazepam		Nifedipine	
Amitriptyline			Doxazosin	
Fluoxetine (daily)			Dipyridamole	
Barbiturates (except phenobarbital)			Ticlopidine	
Thioridazine			Non-steroidal anti-inflammatory drugs	• Indomethacin
Meperidine				• Naproxen
Anorexics				• Piroxicam
Amphetamines			Myorelaxants and antispasmodics	• Carisoprodol
Anti-histamines	• Chlorpheniramine			• Chlorzoxazone
	• Diphenhydramine			• Cyclobenzaprine
	• Hydroxyzine			• Orphenadrine
	• Cyproheptadine			• Oxybutynin
	• Tripelennamine			• Hyoscyamine
	• Dexchlorpheniramine			• Propantheline
	• Promethazine			• Belladonna alkaloids
Chlorpropamide			Ketorolac	
Unassociated estrogens (oral route)			Ergot and cyclandelate	
Thyroid extract			Laxatives	• Bisacodyl
Methyltestosterone				• Cascara sagrada
Nitrofurantoin				• Mineral oil
Ferrous sulfate				
Cimetidine				

The Chi-square (Fisher's exact) test was employed in the statistical analysis as a comparative criteria between groups.

This study was part of Project No. 405/10 (Senile dementia and medications) approved by the Research Ethics Committee of the Irmandade da Santa Casa de Misericórdia de São Paulo.

## RESULTS

The patient population studied had a mean age of 81.5±8.8 years and comprised 29 demented (21 women 8 men) and 21 non-demented (16 women and 5 men) elders, with mean consumption of 5.6±2.9medications/elder - 12 consumers of up to 3 medications (7 D and 5 ND) and 38 consumers of 3 or more medications (22 D and 16 ND).

The most consumed medications not related to treatment for dementia conditions among the demented group (mean of 5.7±3.0 medications/demented elder) were: aspirin, angiotensin-converting enzyme inhibitors, statins, selective serotonin reuptake inhibitors, and vitamins. The most consumed medications among the non-demented group (mean of 5.5±2.9 medications/non-demented elder) were: vitamins, aspirin, calcium carbonate, proton pump inhibitors, statins and alendronate sodium. As shown in Table 2, comparisons revealed no statistically significant differences between the groups. However, a p-value of 0.06 (Yates) was found for vitamin consumption in ND whereas all other p values were greater than 0.06. Use of PIMs for elderly was identified in 14 patients (10 D and 4 ND). No statistically significant differences for PIM use was detected in elderly groups by gender or age.

**Table 2:** Drugs reported by demented and non-demented elderly at first geriatric outpatient clinic appointment.

Drugs	Demented						Non-demented						Total and significance
	**Women**			**Men**			**Women**			**Men**			
	**<80 years**	**≥80 years**		**<80 years**	**≥80 years**		**<80 years**	**≥80 years**		**<80 years**	**≥80 years**		**D+ND = total**
Vitamins	1	4		-	3		5	7		1	-		08+13=21*
Aspirin	2	5		1	2		3	2		-	2		10+07=17*
ACEI	2	5		-	2		1	3		-	1		09+05=14*
Statins	3	4		-	1		3	1		-	2		08+06=14*
PPI	-	5		-	1		2	3		1	-		06+06=12*
Thiazides	3	4		-	1		1	2		-	-		08+03=11*
Levothyroxine	-	5		-	1		2	2		-	-		06+04=10*
β Blockers	1	4		-	-		1	2		1	1		05+05=10*
CaCO_3_	1	1		-	-		3	5		-	-		02+08=10*
SSRI	1	5		1	1		-	1		-	-		08+01=09*
Ginkgo biloba	1	1		-	3		3	1		-	-		05+04=09*
ARB	-	4		-	-		1	1		-	2		04+04=08*
Alendronate	1	1		-	-		3	3		-	-		02+06=08*

D: Demented; ND: Non-demented; ACEI: angiotensin converting-enzyme inhibitors; SSRI: selective serotonin reuptake inhibitors; CaCO3: calcium carbonate; PPI: proton pump inhibitors; ARB: angiotensin receptor blockers/antagonists;

* No statistical significance between main groups (D and ND) and/or their subgroups (p≥0.06).

## DISCUSSION

The mean consumption of medication in the present sample was in line with previously levels reported in other Brazilian studies.^13-15^ Elderly populations often present with more than one disease concomitantly, leading to the simultaneous use of multiple medications^13-16^ Consequently, this patient group is exposed to greater risk of drug interactions and adverse side effects.^1-5,13-16^ The association of three factors, namely, number of diagnoses, drugs in use and PIMs for the elderly, should always be analyzed carefully in daily routine practice involving this age group.^15^ Although beyond the scope of the present study, it is important to bear in mind that drugs typically used in cases of senile dementia - acetylcholinesterase inhibitors, memantine and psychodrugs - also present adverse effects and drug interactions, where their indication/prescription to patients requires prior risk assessment.^17,18^

Medications not directly related to the treatment of senile dementia accounted for the majority of the most frequently prescribed drugs in both groups, with the exception of selective serotonin reuptake inhibitors, taken by around 25% of the demented group and by the older subgroups of both main groups. It is noteworthy that the signs and symptoms of depression can be confused with the clinical condition of dementia in its early phases, constituting one of the main differential diagnoses of dementia syndrome.^17,18^ and a cause of referral to the Geriatric outpatient clinic analysed in this study. Depressive conditions are frequent in older adults and can be attributed to neurodegenerative diseases and to the physical, social and cognitive losses common in this age group.^17,19^

A curious finding in the present sample was the elevated number of elderly in use of vitamins - 42.0% of total cases. Examining vitamin users in the two groups, the proportion of non-demented consumers of vitamins was found to be double that of demented consumers. This result was somewhat expected given that the typical consumer of vitamins is female, elderly, non-institutionalized with a balanced diet, low BMI, high level of physical activity and of schooling.^20^ Items in this consumer profile, such as physical activity and schooling, are also deemed protective factors against developing dementia conditions.^21-23^ Thus, it is evident that the usual consumers of vitamins are those who least need this supplementation, given their lifestyle and health, and may be innocently exposing themselves to collateral effects and drug interactions^24-26^ The same observation holds true for use of the phytotherapeutic drug *Ginkgo biloba*, which has a similar action to aspirin and can potentially intensify the inhibitory platelet aggregation action of salicylate and other non-steroidal anti-inflammatory drugs.^4,27^

The other medications consumed in both groups of the population analyzed are normally prescribed in cases of diseases commonly found in this age group.^1,3,13,15^

The qualitative assessment of the medications was performed using the penultimate version of the Beers-Fick criteria^5^ as opposed to the more recent version^1^ because the latter was only published after completion of the present article and had not been adapted for the Brazilian Pharmacopeia. In light of this situation, Brazilian literature which also applied the same version^5^ was consulted showing that, based on these criteria, the present sample had a lower percentage of elders consuming PIMs compared with the other studies examined.^15,27,28^ Lastly, the high number of users of vitamins, *Ginkgo biloba* and PIMs for elderly seen in this study raised concerns, representing a stand-out factor in the first appointment at the Geriatrics outpatient clinic.

In conclusion, the present casuistic, independently of dementia status, gender, or age group, had similar drug consumption patterns with use of multiple drugs concomitantly.

These results highlight the pressing need for studies investigating drug side effects and/or interactions mimicking cognitive impairments in elderly that stem from the use of medications typically prescribed in this age group.
